# Learning from the COVID-19 Pandemic: Implications from Governance Capacity and Legitimacy

**DOI:** 10.1007/s11115-023-00705-5

**Published:** 2023-03-29

**Authors:** Jonas Lund-Tønnesen, Tom Christensen

**Affiliations:** Department of Political Science, University of Oslo, Oslo, Norway

**Keywords:** Administrative capacity, Coordination, COVID-19 pandemic, Crisis management, Learning, Norway

## Abstract

This paper examines the crisis management learning by the Norwegian government after the COVID-19 pandemic by focusing on types of learning based on the concepts of governance capacity and legitimacy. Using unique interview data with 36 elite administrative and political executives in Norway, the study finds varied learning by the involved actors, and most learning about coordination between ministries and agencies, which are amplified by the lack of knowledge related to analytical capacity. The study contributes to advance the analytical understanding of crisis management learning and provides insight into what a high performing government in the pandemic attempts to learn.

## Introduction

A central issue in crisis management concerns learning from previous crises (Boin, ‘t hart, Stern and Sundelius, 2005). This is a key precondition for building governance capacity and legitimacy for dealing with future crises (Christensen et al., 2016). After governments deal with a major crisis such as the COVID-19 pandemic, many opportunities emerge related to understanding what worked and what did not work in crisis management. The COVID-19 pandemic began as a major health crisis, but eventually developed into a societal crisis after governments across the world introduced measures to deal with the virus. This had major consequences in all parts of society, which provided administrative and political actors with many aspects to focus on in the learning phase of handling this pandemic (Ansell et al., 2021; Dunlop et al., 2020).

To gain a deeper understanding of what central actors involved in crisis management identify, stress and focus attention on in the learning phase, this study connects the concept of learning with governance capacity and governance legitimacy in crisis management. It does this by studying the learning of the Norwegian government after dealing with the COVID-19 Pandemic. Norway is generally seen as a high performer in the pandemic (Christensen & Lægreid, 2020), but that does not mean that all aspects of dealing with the pandemic went smoothly and without difficulties. Decisions were made under great uncertainty, with limited time to act, and unclear consequences of the implemented measures (Lund-Tønnesen, 2022).

While there have already been studies on learning after dealing with the pandemic, this has primarily concerned the initial phases of the crisis (e.g., Boin et al., 2020; Lee et al., 2020; Wolff and Ladi, 2020). At that time, the crisis was mainly about dealing with the virus rather than the far-reaching societal issues. To understand learning after two years of dealing with the pandemic, unique data from 36 interviews with elite administrative and political executives in Norway who were central in the decision-making processes throughout the pandemic is used and analyzed. Based on this, the main research questions are:

- What did the Norwegian Government Learn from Dealing with the COVID-19 Pandemic?
- How can the learning and post-crisis management reflections be understood based on features of governance capacity and governance legitimacy in combination with theories of learning?

## Theoretical Background

### Crisis Management, Governance Capacity and Legitimacy

Crisis management is the systematic use of diverse capacities to avoid, be prepared for, mitigate, make sense of, influence stakeholders perception of, and learn from crisis. The capacities that the political-administrative leadership in general and the crisis management leadership specifically is using can be divided into four types (Lodge & Wegrich, 2014). First, analytical capacity, that alludes to the need for understanding the relationship between means and ends, i.e. what Dahl and Lindblom (1953) label rational calculation. Scoring high on analytical capacity not only improve the likelihood of achieving goals, but also overall saving resources. Regulatory capacity deals with what type of regulations is used and how strict the regulatory measures are defined (Lund-Tønnesen, 2022). These are both crucial under a pandemic. Balanced measures are difficult to achieve, because such measures either are too draconic or too soft, depending on the context. Coordinative measures are about organizing contact and collaboration among relevant actors both inside the public apparatus and between this apparatus and the environment. These measure relate to the fact that crises often are reflecting what is called ‘wicked issues’, meaning issues that is reaching across countries, levels, sectors and institutions (Head & Alford, 2015). Delivery capacity deals with how effectively public authorities are delivering services during a crisis, which is crucial. Two examples from China are fitting. In Wuhan, the authorities managed to organize food delivery to people during extreme difficulties, and the higher authorities delivered a lot of medical services through so-called ‘paired-assistance programs’, meaning programs that provided help from doctors and nurses in other provinces than Hubei (Christensen & Ma, 2021).

Legitimacy deals primarily with how people perceive how the public leaders have dealt with the crisis, which could vary a lot according to type and degree of capacity used, type of actor and context/situation (Christensen et al., 2016). This alludes to what Schillemans (2008) would label horizontal or voluntary accountability, i.e. public leaders are appealing directly to the public, often through diverse media, to influence their perception and therefore their support. Input legitimacy alludes to the quality of the participation of the public during crisis, throughput legitimacy deals with internal processes and coordination, while output legitimacy focuses on the end product, meaning how well the authorities’ handling is perceived overall.

There is a dynamic relationship between governance capacity and legitimacy (Lund-Tønnesen & Christensen, 2023). The ideal is that the capacities used by the government are handling a crisis very effectively, the perception by the citizens supportive and the legitimacy very high. The most problematic combination for the public leadership is few capacities available and overall low legitimacy. However, there could also be high capacity and low legitimacy, or low capacity and high legitimacy.

### Learning

Learning can be understood as “*the ability to detect and correct errors and thereby to improve the functioning of an organization*” (Olsen & Peters, 1996, p. 4). This involves to identify, remember and use procedures and structures that enhance the problem-solving capacity of the government to prepare it for the future (Lodge & Wegrich, 2014). In essence, learning from experience is a substitute to calculative rationality (Levinthal & March, 1993). It is a central part of crisis management (Boin et al., 2005), and the aspirations for learning are often high in contemporary polities and organizations. There does not exist an established theory of experiential learning that can be linked to crisis management (Smith & Elliott, 2007; Antonacopoulou & Sheaffer, 2014). Moreover, there are also disagreements as to what extent governments can actually learn (Stern, 1997). However, one can advance and nuance the understandings of what government attempts to learn in the aftermath of crisis management. This pertains to the use of experience, where the government consults the past to improve governance performance.

Crises are situations that are believed to inspire learning (Boin et al., 2005). Learning from a major crisis like a pandemic is a case of “*learning from samples of one or fewer*” (March et al., 1991). In such instances, experience can guide government to repeat former routines, or function as precondition for flexibility and experimentation that can result in comprehensive adaptation and change (Olsen & Peters, 1996). When dealing with major crisis such as a pandemic, many actors including politicians and bureaucrats are often involved, each with their own beliefs, values, and cognitive biases. Under conditions of high uncertainty and ambiguity as in a major crisis, experience is created and interpreted differently (March et al., 1991). Diverging beliefs, values, and biases can make different actors make different choices when it comes to identifying and searching for lessons to be learned (Rose, 1993). Superstitious learning may occur (March & Olsen, 1975), meaning there is an attempt to learn without knowing or perceiving in a biased way the actual relationship between action (crisis measures) and response from the environment (citizens, the governance apparatus). These are typical barriers to learning after crisis, which might lead to more simple changes in procedures and structures, rather than changes in deeper elements such as core beliefs and values (Smith & Elliott, 2007).

Learning related to governance capacity and governance legitimacy is about which aspects of these two concepts the involved actors detect, draw attention to, and stress after being involved in crisis management. Which aspects of capacities were crucial or problematic? Which aspects of legitimacy proved to be critical and how were their dynamic relationship to the capacities? Overall, the expectation is that because experience may be interpreted differently by different actors such as politicians and bureaucrats, one will see variation in the learning regarding what happened and what is emphasized in the sub-dimensions of governance capacity and legitimacy. Precisely because there are many actors involved – in Norway such as the prime minister, different ministers and ministries, and two professional health agencies – one can expect a significant emphasis on the coordination dimension (Lægreid & Rykkja, 2019; Førde, 2022). This could be about reflections regarding lead agency and allocation of authority between the professional health agencies, Ministry of Health, Ministry of Justice, and politicians.

Furthermore, because there is generally a lack of knowledge about the consequences of such pandemics and analytical capacity is often seen as a fundamental precondition for both governance capacity and legitimacy, one may also expect analytical capacity to be significantly emphasized in learning. In that regard, the expectation is that a positive consensus among health experts about the effects of a crisis measure will lead to consistent learning between bureaucrats and politicians. If health experts disagree, then learning is likely to be diverging. Relatedly, one can expect to see varying focus and interpretations of the effects of regulatory measures, planning and flexibility in the decision-making during the pandemic. This is not to say that delivery capacity is not expected to be subject to learning, which could be for example be related to allocation of vaccines, but with a mega-crisis that affects all aspects of society, some components of crisis management will likely be given prominence over others in learning.

When dealing with a pandemic like the COVID-19 pandemic, the expectation is that involved actors are particularly concerned with output legitimacy, because ultimately it is on performance that they likely believe they will predominantly be evaluated. One may also expect some learning regarding input and throughput legitimacy because this concerns how involved actors reflect upon contextual conditions such as trust and a well-functioning health care system in Norway. Additionally, it can be about reflections on the inclusion of experts in the decision-making forums, but also relate to regulatory capacity about how new rules and measures are developed and communicated in the governance apparatus.

## Methods and Data

This is a single case study of the Norwegian government’s learning after the COVID-19 pandemic. Norway is chosen as a case because it provides a unique opportunity to gain insight into what a high performer in crisis management focuses on and highlights after the crisis (Christensen & Lægreid, 2020). Interviews and documents constitute the data sources. The study uses data from unique interviews with elite administrative and political executives in Norway who were central in the crisis management and decision-making processes throughout the COVID-19 pandemic. The 36 interviewed actors include the former and current prime minister, six ministers, and a number of top civil servant leaders in the Norwegian Institute of Public Health (NIPH) and the Norwegian Directorate of Health (NDH) as well as other relevant agencies and ministries.

The interviews were conducted by the independent Corona Commission in Norway whose mandate was to do a thorough and comprehensive review and evaluation of the Norwegian government’s handling of the COVID-19 pandemic. The interviews were carried out between November 2021 and February 2022 and lasted between 45 and 120 min. They are publicly available for download at the commission’s website and are transcribed in their entirety. The interviews include questions on all aspects regarding handling the pandemic, such as the central crisis management of the government, intensive care capacity, preparedness, vaccine strategies, dealing with the virus specifically, all financial, health and regulatory measures, as well as learning. Learning is an explicit question asked in the interviews, but also implicit in most of the questions.

An assessment was made about what was and what was not relevant to the research questions, as such interviews almost by nature concern ex post assessments about all sorts of things related to dealing with the crisis. The assessment criteria for excluding information were those statements that mainly dealt with general considerations not relevant to the pandemic, and very descriptive information e.g., about what background informants have and what happened at which moment. Everything related explicit to the questions about learning were included, but also implicit questions about what could have been better regarding certain aspects, what worked well and what did not work well, what will be important for future crisis management, decisions made at the time and assessments of those decisions. In the analysis, all the statements concerning learning and retrospective reflections were categorized and analyzed using the software NVivo, based on the description of governance capacity and legitimacy in the theory section. This interview data was supplemented with data from the Corona Commission’s second report. The report is 481 pages long, was published in April 2022 and is a post-crisis evaluation of the government’s crisis management (NOU, 2022: 5).

The disadvantage with using this type of interview data is obviously that the authors could not pose exactly the questions they wanted to, based on the research questions, but had to rely on the questions from the commission. The advantage is that the interviews were both broad in scope and deep, where the main actors had a strong pressure on them to give extensive and honest answers that were reported to the public for scrutiny.

## Results

### The Main Learning Points Regarding Governance Capacity and Legitimacy

The main learning points from handling the COVID-19 pandemic that emerged from interviews with the 36 central actors are summarized in Table 1 below. The overall topics concern coordination, knowledge, decision-making, communication, and crisis management performance, under conditions of urgency and uncertainty, which will be elaborated on in the following.

**Table 1:** Overview of managing the COVID-19 pandemic from a learning perspective

Identified topic for learning	Involved actors	Highlighted learning points related to governance capacity and legitimacy
Coordination of political decision- making in RCU. Administrative coordination by Ministry of Justice (MJ), and Ministry of Health (MH).	Ministers and party leaders. MJ and MH for administrative coordination.	Coordination capacity and analytical capacity focus. Critical learning points identified regarding the political decision-making body and the administrative coordination arrangements. Involved actors appreciated the RCU. The organizing of MJ as lead agency is a central focus point, viewing this as a necessary arrangement so that MH could focus on health-related matters.
Knowledge generation about the virus, and consequences for human health and society.	Primarily NDH and NIPH, but also all actors for their own sectors.	Analytical capacity mainly highlighted, but also coordination capacity. Learning about the lack of knowledge about the virus, and lack of understanding the effects of the measures implemented. Stressed how it is easier to implement measures than to reverse them, also a governance legitimacy concern. Actors reflect on how NDH and NIPH had unclear analytical roles, making them generate the same knowledge, but provide diverging advice.
Crisis decision-making, balance between political and administrative decisions.	Politicians, supported by NDH and NIPH.	Learning about coordination and input and throughput legitimacy. Actors highlight that the separation of tasks between politicians and civil servants worked well, and some recommend this for the future. This concerns involving experts to obtain knowledge, and to legitimize the process. Unclear learning regarding how detailed/strategic politicians should be. Actors prefer that politicians and not civil servants make the final decisions.
Planning and improvising.	Politicians, civil servants in the health sector.	Learning focus related to delivery, analytical and coordination capacity. Disagreement about learning regarding preparation and flexibility throughout the pandemic. Politicians stress flexibility, civil servants stress planning, meaning somewhat unshared learning. Consensus about planning for international cooperation. Lack of plans for dealing with children in crises highlighted.
Internal and external communication of regulatory measures.	Prime Minister and ministers of justice, health, and finance, and NDH, NIPH, DE, police.	Coordination, regulatory and delivery capacity, and throughput legitimacy aspects emphasized. Learning identified regarding the communication of rules internally in the governance apparatus, and capacity problems related to ambiguity of rules and frequent rule changes. Also, externally towards citizens, particularly groups with foreign culture/different language. Multiple actors involved in communicating many rules, making it difficult to be consistent.
Overall view of crisis management performance.	Most politicians and civil servants.	Delivery capacity and output legitimacy learning. Involved actors appear largely satisfied with the performance but consistently insist that there are opportunities for learning and differ in their identified issues.

In the pandemic, there were several formal coordination arenas for the government, and significant cooperation between public organizations, all of which provide important learning points among the informants. In general, the government’s most important regular meetings were the government conferences and cabinet meetings. In addition, the Government’s COVID-19 Committee (RCU) was established in the initial phase of the pandemic. The RCU was a supplement to ordinary government meetings, set up to help ensure that matters related to dealing with the pandemic could be dealt with quickly. Participants in the RCU were fewer than in a normal government conference, and consisted mainly of party leaders, the Minister of Health, the Minister of Justice, the Minister of Finance, and representatives from NDH and NIPH.

Reflecting on these governmental coordination arenas and specifically the RCU, the politicians and civil servants involved assert that this was a useful body that allowed civil servants to understand the overall political considerations that had to be made, and politicians to gain insight into health assessments and health consequences of the coronavirus. For example, the former Prime Minister said that:

*“We benefited greatly from hearing rather unfiltered reports from NDH and NIPH… it gave us a common understanding… I think it worked very enlightening for everyone” (Former Prime Minister).*

Involved politicians appreciated a decision-making arena where quick decisions could be made, and some pointed out that with the inclusion of NDH and NIPH they had a better information base for the political decisions. The Minister for Foreign Affairs mentions that this could be a disadvantage also, because too much emphasis might be placed on health-related matters because the health authorities have a lot of power when they are the providers of relevant information. Moreover, the director of NIPH said that the RCU was useful and efficient, but also time-consuming. When asked if she would recommend bringing health agencies into the government decision-making processes in future crises, she said:

*“I must think through this answer… I personally spent a lot of time with the government. I am generally very positive about it, but have not thought through it systematically whether it is a mechanism that I would recommend or not….” (Director of NIPH).*

Overall, in the interviews, the involved actors primarily highlight the positive aspects regarding the *political* decision-making and coordinating bodies. Relatedly are also the *administrative* coordination arrangements and learning about the function of this. The Ministry of Justice was the lead ministry in dealing with the pandemic and was responsible for the administrative coordination at the ministry level, which might seem somewhat perplexing because of the crisis’ health focus. However, existing sectorial structure in Norway meant that it was the individual ministries who were responsible for conveying and following up guidelines from health authorities in their own sector, and if necessary, establish additional routines.

In addition, the Ministry of Health was responsible for everything related to health and infection control, meaning vaccination, testing, and coordinating health matters in municipalities. In essence, the Ministry of Justice made the rules, and the Ministry of Health justified those rules. According to the Minister of Justice, this was a division of labor based on capacity and what made practical sense. The distribution of these roles is interesting in a learning perspective because it is one of the main structural innovations established in the pandemic. The Minster of Health said that:

*“It would have been completely impossible for the Ministry of Health to be the coordinating ministry over time when the measures to reduce infection to such a large extent led to a crisis in other parts of society” (Minister of Health)*.

This is because the Ministry of Health is set up to deal with a health crisis, not a national crisis, according to the minister. Furthermore, he compares the current ministerial structure to the structure that existed for years before the pandemic, and thinks that in the previous structure it would have been difficult to be efficient and have a good decision-making structure:

*“It is also a learning point to look at the arrangement we had before the pandemic with two ministers in the Ministry of Health and two ministers in the Ministry of Justice” (Minister of Health).*

The minister believed this would have been extremely demanding for the coordination of the pandemic in both ministries and follows that up with:

*“My advice to future prime ministers is that that arrangement can work in normal times, but not in times of crisis” (Minister of Health).*

These reflections about the organization of the coordinating ministry and the role of the Ministry of Health are supported by several ministers and civil servants. However, it is worth mentioning that the Corona Commission believes that this way of organizing contributed to divide and weaken responsibility (NOU, 2022: 5, p. 452).

### The Relationship Between Administration and Politics Under Uncertainty

Throughout the pandemic, health agencies provided their professional advice to politicians who then made the decisions. In retrospect, it is unclear for informants (1) what type of decisions politicians should make, and (2) what influence the two health agencies should have in these decisions (Askim & Christensen, 2022).

Regarding the second point, both NDH and NIPH extensively highlight the difficulties with the overlapping work that arose during the pandemic. Although actors from both agencies believe their own agency’s role is clear, the Assistant director in NIPH said that:

“*There have been several rounds of attempts to clarify the boundary tasks between the agencies and it is probably still interpreted a little differently*” (*Assistant director, NIPH*).

Furthermore, the assistant director in NDH stressed that managing and interpreting the Infection Control Act is one of the core functions of the NDH, but during the pandemic also NIPH made interpretation of the act. In the same way, providing professional advice about infection control is NIPH’s task, but NDH also did this, according to the assistant director of NIPH, so overlaps and grey zones were imminent. As the uncertainty of the pandemic in some ways decreased, these roles were partly adjusted to coordinate the advice to the ministry, indicating increased learning over time during the pandemic.

The Minister of Health acknowledges that there were disagreements between NIPH and NDH. While NIPH did not always agree with the decisions of the government, the Minister still believes this way of making decisions, where the politicians make the final decision, is the best way to deal with the pandemic:

*“I believe this model is the right one, and I am worried one … will pay too much attention to the challenges with this way of organizing, so that one believes another way of organizing would have been without problems.” (Minister of Health).*

The former Prime Minister echoes the relevance of this distinction between decision making by civil servants and by politicians by stating that:

“*It is the politicians who… must be held accountable… and [who] have the legitimacy to make decisions” (Former Prime Minister).*

This is the core lesson from dealing with the pandemic that she highlights. However, this arrangement led to the government having to make many detailed decisions. Concerning what type of decisions the government should make, there is disagreements between the informants in retrospect. For example, the Minister of Foreign Affairs believes there were very many urgent matters in the RCU and the government meetings, and that one of the learning points is about how this came to be. Many processes happened so fast that there was neither time enough beforehand nor during the process to design a working set of regulations, she tells. This meant that regulations in some cases where unfinished when they were presented to the public:

*“I think one of the lessons we should have, is that we should be better at sorting [matters] in such a way that not everything is urgent” (Minister of Foreign Affairs).*

The minister believes that higher priority should be given to what type of decision government does and does not make, which civil servants involved in the RCU also recognize. In their evaluation, the Commission finds that a very high number of decisions about details were made by the executive government. As an example, they discussed and decided how many participants should participate at specific events and what distance they should hold (NOU, 2022: 5, p. 450). However, the former Minister of Finance appears to believe the opposite:

*“I still believe there is great value that the government … had the overall view and detailed knowledge … Although a government must have an overall responsibility, the challenges are often in the details” (Minister of Finance).*

This minister believes that it was not problematic that the government and the central actors in the RCU made detail decisions. The Minister of Justice and her state secretary also seem to think that the detailed approach was necessary. Thus, one can see variation in what is highlighted in terms of learning when politicians look back in retrospect.

### Uncertain Knowledge and Effects

Another central learning point relates to the lack of knowledge about the virus and the uncertainty of the effects of crisis measures. Almost all the informants emphasize that they wish they had more time to make decisions. At the outset of the pandemic there was lacking knowledge about the properties of the virus from an epidemiological standpoint, which had implications for how to handle the consequences of the virus and the measures. The Director of Health said in the interview that:

*“One of the important learning points in the aftermath of this pandemic is that we as a nation should align ourselves with a better system for assessing the consequences across sectors. It is obvious that we have had a lack of knowledge about the effect of the individual measures” (Director of Health).*

He believes that the government as a whole only has a reasonable overview of the overall effect of measures. Although the government gained more knowledge throughout the pandemic regarding consequences for mental and physical health, work, and the economy, it is not sufficient according to the director. The same is highlighted from another civil servant, who says that they need more knowledge of the consequence of introducing measures in one sector on other sectors.

An additional key learning point is about the consequences of measures that affect children and young people. When asked which groups the informants were particularly worried about in relation to the pandemic, almost all the 36 interviewees who were asked mentioned this group. Civil servants in the Ministry for Children and Families and the Directorate of Education (DE) emphasize that considerations of the proportionality of measures should have been made more explicit than what they were. Regarding the date when the draconian regulations were implemented, the head of DE said in the interview that:

*“If we look back to the days before March 12, 2020, had we known what we now know, we would have recommended not to close kindergartens and schools” (Director of DE).*

Informants in these organizations express that it was very difficult to reverse strict measures once they are implemented, saying that it is:

*“… almost more difficult to reopen than to close… you have had to be particularly confident about why opening is right and base it on knowledge”* (Director of DE).

This concerns to what the existing crisis plans entailed, and what time perspective was assumed for the measures. Interestingly, the NIPH quickly saw that the pandemic likely could last for several years, while politicians do not seem to have had this perspective. For instance, the Minister of Foreign Affairs, said that “*some of the measures were not planned to last as long as they did*”, which had implications for how the measures eventually were designed. Relatedly, there are several reflections of learning regarding the preparedness for a major crisis like this. First, the director of NIPH emphasized the importance of existing plans:

*“…Where we have had plans and those plans have been put to use … there we have succeeded better than where we have to improvise. We have also often succeeded in areas where we had to improvise, but more often with solution that will not last” (Director of NIPH).*

Furthermore, on question about what the most important learning points are, the Director of NDH agrees with the director of NIPH by saying that “*it is about being prepared*”. He believes there is a need to revise existing plans, not only regarding pandemics but also other health crises. This is important for the supply of medical equipment, according to the director.

These points are supported by the director of the Western Norway Regional Health Authority who believes that because of the security situation in the world, Norway is too dependent on Asian countries to produce medical consumables and should join with EU to produce these. The Minister of Health also agrees that an important learning point is that Norway should secure strong cooperation in Europe. He also does not view it as realistic for Norway to have a national preparedness system that will ensure access to vaccines.

What is more, the importance of flexibility is highlighted by some politicians. For instance, the former Prime Minister says that Norway had originally only planned for a pandemic influenza. However, because it became a different type of pandemic impacting all parts of society, she said that “*we have to have flexibility*” and that “*You have to remember that when you face such situations there is never one template*”. Moreover, the Minister of Foreign Affairs says that it is with the combination of planning and flexibility that the recurrent fluctuations of infection can be dealt with. Reflecting on the pandemic, she believes there is a need for improvisation, and points out that:

*“I think we are better prepared to handle the next crisis… When we look at the plans and things we can do better, it must not tip over in such a way that we restrict crisis management according to a certain template, which means that we would lose the ability to improvise” (Minister of Foreign Affairs).*

### Internal and External Communication

Communication of rules and measures internally and externally is one more central learning point after dealing with this pandemic. External communication with the public is something that many interviewees highlighted and were involved with. Sense-making and meaning-making at the start of the pandemic in Norway was clear (Christensen & Lægreid, 2020). However, throughout the pandemic, communication became complex, unclear, and unnecessarily complicated, the assistant director in NDH tells. In some cases, the information about measures were published at press conferences before the details about the regulations were ready. The director of NIPH acknowledges the inconvenience of this, and that the regulations should preferably be clear so that they can be complied with.

Several actors from NIPH, NDH and many ministers at various points in time communicated to the public what the government had decided. The Prime Minister, Minister of Health and Minister of Foreign Affairs highlight this as problematic, especially that the agencies were heavily involved in this, and that they disagreed publicly. At the same time, the Prime Minister emphasizes that it was important to have them at press conferences to show credibility and the medical reasoning for the decisions. Looking back, the Minister of Foreign Affairs believes that there was poor internal communication within and between NIPH and NDH, which meant their message was sometimes undermined. the Minister of Health agrees with this, but says that:

*“The communication challenges towards the population are not large enough compared to the advantages it has. I believe that the population’s trust has increased because there were discussions and different assessments” (Minister of Health).*

We thus see a combination of a complex set of rules and measures that must be complied with and enforced, and many actors who communicate this. Overall, it seems to be that politicians and civil servants were clear regarding overall strategies and goals, but more unclear regarding specific measures and evaluations in their communication to the public.

### Analysis of the main Learning Aspects of Governance Capacity and Legitimacy

#### Learning About Coordination Capacity

Learning related to coordination capacity is one of the central learning points that emerge in the interviews, as highlighted in Table 1. Overall, it seems that the involved actors believed the temporary RCU body worked well, because they think it allowed them to make quick decisions under uncertainty.

In their assessment of the lead ministry, the Ministry of Justice, informants generally view this as a beneficial arrangement, focusing attention towards its nonhierarchical facilitation role (Lodge & Wegrich, 2014). This is rather paradoxical, since the Minister of Justice was somewhat in the background in the press conferences and media generally, and the Minister of Health was very much running the show due to the health focus of the regulatory measures. So, it was a discrepancy between formal and actual accountability structures (Christensen & Lægreid, 2020).

Moreover, aspects of vertical coordination are viewed as quite challenging. Municipalities were often only informed about the implemented measures after public press conferences. There seem to already be changes instituted regarding this by the new government after October 2021, where municipalities are now informed about measures ahead of their announcements. In addition, the new government continued with the model of one Minster of Health and one Minister of Justice and Public Security, which the previous Minister of Health recommended. These are admittedly relatively straightforward structural actions, yet simple forms of learning one can expect after a crisis (Smith & Elliott, 2007).

#### Learning About Analytical Capacity

Learning with reference to *analytical capacity* is a central learning point informants reflect upon. Knowledge about what to do has been difficult to generate, which makes it difficult to learn. Civil servants highlight the lack of knowledge about the virus itself and the effects of the measures. One can interpret this as learning that relevant actors only have an inkling about what to do and how to do it, essentially learning that learning is bounded (March & Olsen, 1975). One paradox with this was that rather early extensive studies were done in China, but this knowledge was not much used in the West, for example on the limited role children played in the first phase of the pandemic (Christensen & Ma, 2021). This seems to indicate a biased and trust-based search process seen from the experts (cf. Cyert and March, 1963).

Moreover, what actors in NDH and NIPH as well as politicians note is that when there is a lack of knowledge about a pandemic and the effects of the measures, the professional health agencies end up working in parallel about the same things (Askim & Christensen, 2022). Thus, when two somewhat similar professional agencies face major uncertainty with a lack of analytical capacity, coordination problems become apparent.

Time is an analytical capacity concept stressed by almost all informants, which relates to the lack of organizational slack to make decisions and the simultaneous increase of workload, in the pandemic (cf. March, 1994). The findings indicate that politicians disagree about what type of decisions to make in times of great urgency and uncertainty, meaning that such situations do not lead to similar interpretations and learning, making it difficult to distinguish success from failure and learn for future crisis management (March & Olsen, 1975).

Furthermore, in terms of learning about planning, there seem to be differences between politicians and administrative leaders. The former notes the significance of flexibility, the latter the significance of planning. One can understand this because they engage in different roles in crisis management. Moreover, civil servants generally have longer careers than politicians, which might indicate that they are concerned with sustaining existing structures and beliefs and working within these structures (March et al., 1991). Hence, their focus was more on what is called exploitation, in contrast to exploration that the politicians favored (cf. Levinthal and March, 1993).

#### Learning About Regulatory Capacity

Administrative leaders from the health, child welfare, and police sections are all concerned with learning related to regulatory capacity, and the difficulties regarding the ambiguity of the rules and the frequency of rule change. Politicians, who are mainly involved in deciding the rules, do not focus much on this, which is expected as actors tend to focus attention at issues in their spatial and temporal neighborhood that concern their own problem-solving capacity (Levinthal & March, 1993).

One of the obstacles of learning was that the executive politicians opted for a standardized strategy in most of the pandemic, even though it was obvious that the pandemic was biased in its spreading and therefore it should probably have been a differentiated regulatory strategy. In the beginning of the pandemic, main actors were unison and the public very much accepted the regulatory measures. But, as the pandemic went on, it was more of a public debate about the logic of some of the measures. The regulatory structure did not align with the structure of the pandemic and one reason could be a fear for even more lack of knowledge with differentiation and also more pressure on the politicians to please certain groups.

One can also say that problems with analytical capacity and coordinative capacity could undermine the regulatory capacity (Sajadi & Hartley, 2022). If experts say that they do not really know or are disagreeing over measures, it is difficult to install effective regulative measures. Ambiguity in goals and time pressure likely did not help either (cf. March, 1994).

#### Learning About Delivery Capacity

Learning about delivery capacity concerns handling the crisis, especially regarding essential services (Lodge & Wegrich, 2014). Informants generally appear to be largely satisfied with the performance of managing the pandemic in Norway in this respect, even though there has been a knowledge deficit. The political and administrative leaders claimed that most of the regulatory measures were necessary, even though it created some problems for people regarding movements, access to some types of stores, access to restaurants/bars, etc. Overall, people felt the collection of measures worked, even if the effect of the individual measures cannot be isolated. For all governments, dealing with this pandemic is an entirely new experience, produced by a complex set of factors in a non-repetitive setting, leading to difficulties of evaluating success (March et al., 1991). The involved actors’ satisfaction may be due to the low infection numbers in Norway compared to other countries, and that general political trust has been maintained, which may give rise to the construction of causal beliefs (March & Olsen, 1975).

#### Learning About Governance Legitimacy

Regarding input legitimacy, learning is clear for some aspects. Civil servants and politicians seem to appreciate that Norway is a well-functioning democratic society (Christensen & Lægreid, 2020). Many decisions in the pandemic were consciously made by politicians, as opposed to only by civil servants, implying that the regulatory measures stem from the people, and not only from non-elected officials. Both politicians and civil servants compare this way of making decisions to Sweden’s approach, where it was not the politicians who made the major decisions, and state that they prefer the Norwegian way. Learning in this case is consistent between informants because they believe that this system provides legitimacy. Nevertheless, it is puzzling that despite the satisfaction with the system, politicians should not have had to make so many detailed decisions as they did. This may indicate that they were gradually more worried about legitimacy loss and being held accountable.

Moreover, the inclusion of actors from civil society in the policy processes can be seen as a part of governance legitimacy but is not reflected upon by any of the interviewees. There is only learning about this in relation to individual policies, for instance the digital contact tracing app (Lund-Tønnesen & Christensen, 2023). As the Norwegian government was quite paternalistic, the inclusion of citizens in the policy process, the use of citizens-driven initiatives or digital-based reforms as in other countries (see Baniamin, 2021; Bawole and Langnel, 2022), could have increased legitimacy for some of the individual measures.

Learning related to throughput legitimacy concerns processes within the governance apparatus (Christensen et al., 2016). Administrative leaders and experts were strongly involved in the crisis decision-making forums, which was important for the legitimacy of the decisions, something that several politicians highlight as a critical learning point. Moreover, the inclusion of experts in communication to citizens was important to show legitimacy and credibility for what the government was doing (cf. Cairney and Wellstead, 2021; Vu, 2021). This indicates learning regarding the importance of conveying what goes on in the “black box” of governmental decision-making.

In addition, throughput legitimacy concerns internal communication of regulatory measures within the government apparatus. If street-level bureaucrats like the police are not familiar with rules, it is axiomatic that it is impossible to enforce them. During the pandemic, the police created support structures for the frontline officers to assist the interpretation of rules, but the police as a whole ended up doubting whether these rules were constitutional or not. It was also problems for local authorities to know about the constant stream of changed or adjusted regulations – it was over 300 changes in the national pandemic rules - and it was conflicts over the power of the lead local doctors based in the infection protection act. Relevant actors emphasize this point, along with the need for more time to interpret rules as a critical learning point.

Third, there is also interesting learning regarding output legitimacy (Christensen et al., 2016). Just as with enforcers, it is axiomatic that rules can only be complied with, and by extension viewed as legitimate, if citizens know of them. Informants stress this aspect as an important learning point. Output legitimacy also relates to learning over time during a crisis, i.e., intra-pandemic learning (Lund-Tønnesen & Christensen, 2023). Citizens’ responses to output measures work as mechanisms that feed back into institutional practices (Easton, 1965). As some civil servants stress, if the government learns the effect of measures and of the intended rules, there can be fewer measures overall, the measures can be less intrusive, and more targeted. This could lead to higher support for the government and higher compliance to regulatory measures (Vu, 2021). In this way, there is unrealized learning capabilities about the dynamics between different forms of governance capacity and the general output legitimacy of managing the crisis. Finally, as previously mentioned, all informants appear to be largely satisfied with the performance of managing the pandemic in Norway, but there are many inconsistent lessons identified as to how future pandemics should be dealt with.

## Conclusion

This study set out the answer the questions: *what did the Norwegian government learn from dealing with the COVID-19 pandemic? How can the learning and post-crisis management reflections be understood based on features of governance capacity and governance legitimacy in combination with theories of learning?* Using data from 36 unique interviews, the study provides a comprehensive account of what administrative and political executives in the Norwegian government learned from dealing with the pandemic. Additionally, it demonstrates how one can understand learning from a crisis based on the concepts of governance capacity and legitimacy.

The main finding of the study is that coordination, even in a generally well-functioning government, is challenging and offers many lessons to be learned (Lægreid & Rykkja, 2019; Førde, 2022). Analytical capacity is also highlighted as a central learning aspect, together with the communication of regulatory measures. In the learning phase, civil servants and politicians focus on and stress different issues (Rose, 1993). This is due to the involved actors having different interests and interpretations of what was important in managing the pandemic, where they often emphasize learning aspects related to their own problem-solving capacity (Levinthal & March, 1993). For future research it will be vital to also study pandemic matters more related to learning in public policy (Dunlop et al., 2020). This could be about alternatives to vaccination in early stages of managing pandemics (Sajadi & Hartley, 2022), in both high capacity and low capacity governments.
